# Increasing perceived health and mental health literacy among separated refugee Ukrainian families with urgent needs occasioned by invasion—a group intervention study with participatory methodology in Sweden

**DOI:** 10.3389/fpubh.2024.1356605

**Published:** 2024-05-09

**Authors:** Solvig Ekblad, Oksana Gramatik, Yuliia Suprun

**Affiliations:** Cultural Medicine Research Group, Department of Learning, Informatics, Management and Ethics (LIME), Karolinska Institutet, Stockholm, Sweden

**Keywords:** refugees, EU’s mass refugee directive, health promotion, trauma, stress, communication, separated families

## Abstract

**Background:**

With the increasing numbers of refugees from Ukraine affected by the ongoing war, there is a high risk of trauma-related stress due to low local health and mental health literacy care. Perceived good health is a human right. Earlier studies show that intervention for refugees can reduce and prevent post-migration stress and anxiety. The present explorative study aimed to investigate the feasibility and effectiveness of a short, trauma-focused group intervention (in Swedish “hälsoskola”) for Ukrainian-speaking refugees (EU’s mass refugee directive). This intervention was part of an ESF project aiming to get the subjects closer to the labor market in Västernorrland County, Sweden.

**Materials and methods:**

A mixed-methods design, a participatory methodology, and an evaluation were used. Data were obtained with a short questionnaire in Ukrainian. It included a visual analogue health-rating scale, an observation, and an oral evaluation in groups. For practical and ethical reasons, there was no control group. Each group met five times for 2 h, a total of 10 h excluding pre- and post-assessment. There were eight sets of five group sessions per set, a total of 40 sessions. Brief initial and concluding breathing exercises sought to reduce stress among the participants.

**Results:**

Baseline data were obtained from 99 participants, mostly females. Data gathered directly after the group intervention from 57 participants who had filled in both the pre- and post-questionnaires showed that (1) perceived anxiety/stress was significantly reduced (chi-2 25,53, df = 4, *p* < 001). (2) The participants showed significantly better perceived health as assessed on a visual analogue scale (average change from 63.16 to 71.18, *p* < 0.001). This result was supported by the participants’ questions, which were qualitatively evaluated using thematic content analysis. Five general themes stemmed from questions raised in dialogue with the participants plus observation with the respective local expert. The participants received answers to their questions, and their perceived negative attitudes to authorities changed to more positive ones.

**Conclusion:**

By dialogue between the participants’ needs of knowledge and direct answers by the local expert, respectively, was feasible and effective as they perceived trust and health and mental health literacy increased. Implications for primary prevention are discussed.

## 1 Introduction

Several studies have shown that a prolonged asylum process not only increases the risk of ill health but also increases the risk of failed integration. This, in turn, can lead to segregation, exclusion, and mental illness (1). The ADAPT model (2) recognizes five main psychosocial posts, which may be disordered by conflict, separation, and displacement, i.e., systems of safety and security, interpersonal bonds and networks, justice, roles and identities, and existential meaning and coherence. These posts are stable in societies with peace.

Asylum seekers have specific healthcare needs; however, contextual and structural factors such as barriers to work, housing, and healthcare negatively affect their health and wellbeing. There have been insufficient efforts to address these needs in the reception programs (3).

The World Health Organization (4) report “Mental health of refugees and migrants: Risk and protective factors and access to care” identifies several risk factors such as trauma, violence, discrimination, exclusion, language barriers, and limited socioeconomic opportunities. The report also addresses several protective factors, including access to educational and work opportunities, social support, and cultural connection.

Research on the course of post-traumatic stress disorder (PTSD) after a single potentially traumatic experience shows that about one-third of subjects develop PTSD (5), but many probably have several traumas, so the number can be higher. Regardless of definite numbers, international literature underlines the urgent need for culturally sensitive interventions early in refugee reception centers in host countries to reduce and prevent acute and chronic illness.

A systematic review and meta-analysis of the efficacy and acceptability of psychosocial interventions in asylum seekers and refugees shows that most evidence-supported interventions are based on cognitive behavioral therapies with a trauma-focused component (6). A pilot study in a German reception center with psychotherapeutic group intervention using imaginative stabilization techniques for traumatized male refugees offers a promising and feasible approach to treating refugees in unstable reception-center settings. Existing international literature on evidence-based programs (especially trauma-focused interventions) for new groups of refugees shows research gaps according to existing needs and a lack of healthcare resources. It is also unclear how the study intervention tries to respond to existing needs (7).

Too little is known of how group interventions which reduce the newcomers’ distress and anxiety symptoms while strengthening their internal resources and increasing their emotional stability could be developed as routine, and how they could be available in healthcare for asylum seekers and refugees with mental illness due to trauma.

Refugees have been identified as groups having lower health literacy, which may affect healthcare-seeking behavior (8). Health literacy “entails people’s knowledge, motivation and competencies to access, understand, appraise, and apply health information in order to make judgments and make decisions in everyday life concerning healthcare, disease prevention and health promotion for themselves and those around them[(9), page 7].

One especially vulnerable group is the Ukrainians who according to the EU’s mass refugee directive have received temporary protection in the European countries. The majority are women and children, while the husbands are soldiers in Ukraine. The Russian full-scale invasion of Ukraine on 24 February 2022, has led to one of the largest refugee crises in recent history. Javanbakht (10) addresses the following practical first steps in a host country to reduce the long-term impact of trauma and stress on refugees’ mental and physical health and functioning. Steps include mental health first aid, education in mental health, and how to navigate the healthcare system. Furthermore, the literature shows a risk for separated couples to reach the exhaustion stage according to Selye’s general adaptation syndrome (11), the consequence of which may be the divorce of the spouses. There is evidence that war trauma influences parenting behavior and women have a 2–3 times higher risk of developing post-traumatic stress disorder (PTSD) compared to men (12).

The effect of such stresses may increase conflict among spouses, family members, children, and close friends. Family coping resources are diminished by long forced separation, physical injury, and mental illness (e.g., negative thoughts). Children will forget their separated parents (often their father), and this situation risks destroying their development and trust. A study among transnational families shows that loss of social support and family cohesion arises when one of the parents migrates (13). Transnationalism relates to the way individuals, through migration, live in new social worlds, and construct social networks beyond their home country. The rapid transformation from one to the other may increase the risk that refugees become poor and marginalized, as well as increasing stress and worries from pre- and post-repatriation, with negative impacts on mental health and wellbeing. Lack of access to resources, and poverty, may be barriers to healing of past traumas (14).

Enhancing health and mental health literacy is of the utmost importance to improve the health and wellbeing of separated families. Unfortunately, there has been limited attention to investigating the means of improving asylum seekers’ and refugees’ health and mental health literacy through the perspective of families. Family health provides a holistic perspective on the overall wellbeing of the family unit and is an interdisciplinary and complex concept that involves multiple factors.

Given these potential risks of developing social, marital, and health problems among separated Ukrainian couples, it is important to understand protective factors that may reduce these outcomes. However, to our knowledge, there is yet no literature on perceived health and mental health literacy for Ukrainian refugees. During the 10 years before the COVID-19 pandemic started in early 2020, culturally tailored participatory health promotion group intervention with trauma focus (in Swedish “*hälsoskola*”) for newcoming mainly Arabic-, Dari-, and Somali-speaking women with war experiences showed promising results (8, 15–19). Pre–post-evaluation showed that the participants felt less stress and fear about the future and perceived higher health and mental health literacy. From clinical experience in general, primary prevention is today neglected, especially for vulnerable groups in need of care and poorly organized, though the issue is being addressed in many time-limited projects.

### 1.1 Hypothesis

This explorative study stems from the realization that there is a paucity of data on the health and mental health literacy of Ukrainian refugees (EU’s mass refugee directive) in Sweden and postulates that a short trauma-focused group intervention (hälsoskola) for traumatized Ukrainian-speaking refugees reduce their perceived stress and anxiety and have a positive impact on their perceived health.

#### 1.1.1 Aim

For these reasons, the present explorative study aimed to investigate the feasibility and effectiveness of a short trauma-focused group intervention for traumatized Ukrainian-speaking refugees, the majority of women, sheltered in five municipalities in Västernorrland, according to the EU’s mass refugee directive.

## 2 Materials and methods

### 2.1 Context

This study was part of a social fund project (Care-Ukrainare i Härnösand 2022/00433, www.invandrarindex.se) and was performed in five municipalities in Västernorrland: Härnösand, Sollefteaå, Sundsvall, Ånge, and Örnsköldsvik.

STROBE Statement—checklist (20) was used as a guide to reporting.

### 2.2 Procedure

We used the ADAPT theoretical model developed by Silove (2) and a participatory methodological approach (21) according to the five themes (Table 1). The first author trained and supervised the second and third authors about how to plan, perform, and follow up the group intervention (22). After that, we informed the local coaches and experts, on-site and/or online, weekly. At the beginning of the project, the three authors had a workshop with the local experts and coordinators regarding differences and similarities between Ukrainians and Swedes (see Table 2 for examples).

**Table 1:** Themes of intervention sessions led by different local experts (could be in different order locally and according to participants’ length of stay).

Theme	Local experts
1.	Stress, recovery, and repatriation stress (by the three authors)
2.	Human rights issue (local police) or Migration Agency (local assistant)
3.	Asylum health (local nurse) and student health (local school nurse)
4.	Work options (the Employment Agency)
5.	Social welfare (local social secretary)

The intervention comprised a 2-h session/week for 5 weeks, totally 10 h excluding 1-h information and consent and 1-h evaluation.

**Table 2:** List of examples, experienced differences, and similarities in values between Ukrainians (U) and Swedes (S) before starting the short intervention.

Differences	Similarities
Email usage: U prefer phone apps (WhatsApp, Telegram, and Viber) for personal and official communication, while S uses personal email more often.	Education: Both U and S attach great importance to education. They have well-established educational systems and both countries have high literacy rates.
Cultural diversity: U may have less experience with cultural integration compared to S, leading to communication difficulties.	Love of nature and animals: Both U and S deeply appreciate nature. Both countries have much natural beauty, and many people enjoy outdoor activities such as hiking, camping, and fishing.
Communication style: U tend to be more direct and straightforward in communication, while S communicates more indirectly and politely.	Strong work ethic: Both U and S are known for their strong work ethics. They take their jobs seriously and strive to achieve their goals.
Punctuality: U may be more relaxed about timing, while S highly value punctuality.	Respect for the boundaries of another persons: Both U and S deeply value personal boundaries and the boundaries of others.
Socializing: U place a high value on socializing and spending time with family and friends, while S may prioritize personal space and privacy.	Communication with colleagues: Both U and S like to go to coffee/breakfast to chat with colleagues.

During a monthly information meeting in each municipality, potential participants received written and oral information in Swedish, Ukrainian, or Russian about the group intervention, in which participation was voluntary. Those who were interested signed consent forms. A local Swedish- and Ukrainian-or-Russian-speaking coach coordinated the intervention in each municipality. The sessions were in Ukrainian and Swedish, sometimes Russian as the participants had Russian as their mother tongue. Each group included approximately 10–12 participants, but for practical reasons outside our control, there could be fewer or more. A group of 12 deaf participants worked with an interpreter in Kyiv on Zoom (these participants knew sign language in Russian but not Ukrainian).

The three authors usually attended the first of the five weekly sessions to inform and start up. The authors then participated online, as the geographical distance between Stockholm and Härnösand was 430 km and there was no project budget to go by train every week. When being in Härnösand car transport was necessary to go even further during several hours to the other four municipalites. The second and third authors noted questions from the participants and answers from the local experts.

The groups were closed to increase trust during each session. There were eight basic principles: (1) The interpreter had a duty of confidentiality and translated everything said in the room. (2) Everything said in the room stayed in the room. (3) Mobile phones were switched off. (4) Concentration on the here and now and feeling mentally good. (5) Religion and politics as well as economy and housing discussed in other contexts. (6) Hands were raised for questions and comments. (7) “It’s a closed group-no one comes or goes.” (8) No information was given to authorities (privacy was the main policy, but there were several exceptions such as reporting obligations under Swedish legislation governing professionals).

Each session included a pause for refreshments. At the beginning and end of each session, there was a short breathing relaxation exercise supervised by the second and third authors to reduce participants’ stress. The first author has included breathing exercises in the earlier mentioned short group intervention (hälsoskola) toward newcoming refugees and with positive results; reduced perceived stress and anxiety. Perciavalle et al. (23) have shown results obtained from research that support the possibility that the deep breathing technique is capable of inducing an effective improvement in self-reported evaluations of mood and stress and objective parameters (e.g., heart rate and salivary cortisol levels).

### 2.3 Study design

The design was based on Ranganathan and Aggarwal (24). The study had a mixed-methods design (8). The measures had been developed within earlier projects performing short group intervention (hälsoskola) by the first author and co-authors. Yet no reliability and validity measures have been performed due to time-limited projects and have been explorative for different language groups.

The following assessment points were defined:

- *Baseline measurement of the severity of anxiety/stress symptoms related to perceived health*: The anxiety/stress question had three response options (none, to a certain extent, and to a great extent) with subsequent questions with open-response options: Before: if you feel anxiety/stress, what do you feel is the cause of your anxiety/stress? If you feel anxiety/stress what do you usually do to feel better? Further, estimated perceived health was assessed on a visual analogue scale where 0 represented the worst possible perceived health and 100 the best.
- *Pre–post perceived health and mental literacy:* If you feel anxiety/stress, what do you experience after the group intervention are the causes of your anxiety/stress? What knowledge and tools have you gained during the group meetings to feel less anxiety/stress and feel better?
- *Direct after, follow-up symptom-severity measure of perceived anxiety/stress*: We used the same three-option question regarding anxiety/stress as for the baseline measurement to assess symptom reduction. Estimated perceived health was assessed again on a visual analogue scale where 0 represented the worst possible perceived health and 100 the best.
- *Group dynamics:* Group dynamics were operationalized by estimating activity by the participants who asked questions and made comments in each meeting.
- *Follow-up with individual psychosocial support online under supervision*: The results will be reported elsewhere.

### 2.4 Participants

#### 2.4.1 Inclusion criteria

Ukrainian people residing for a fixed period in the above five municipalities in Sweden according to the EU mass refugee directive. They were not allowed to seek asylum. They could communicate with relatives daily but at a distance. All these issues were special for this refugee group. At the start of the project, the target group had permission to stay until 4 March 2023. This was then extended to 4 March 2024 and subsequently 4 March 2025.

100 Ukrainian participants, mostly women, participated between 1 October 2022 and 30 September 2023.

#### 2.4.2 Exclusion criteria

Newcomers from other countries, and Ukrainians who had not arrived under the mass refugee directive and lived in other municipalities.

### 2.5 Translation and back translation as a culturally adapted guideline

The information, consensus, and pre- and post-intervention questionnaires were translated from Swedish into Ukrainian or Russian and back according to WHO’s recommendation (25). The material was also culturally adapted and discussed among the three authors.

### 2.6 Quantitative and qualitative analysis of independent and dependent variables

All statistical analyses used the Statistical Package for the Social Sciences (SPSS) program version 28.0.1.1. Demographic variables and baseline characteristics were analyzed using descriptive statistics (frequencies, means, and standard deviations). *Independent* var*iables*: age, number of children, number of months after arrival, number of moves in Sweden for reasons given by the Swedish Migration Agency, country of birth, mother tongue, status, work in Ukraine, and work in Sweden.

*Dependent variables*: The anxiety/stress question had three response options (none, to a certain extent, and to a great extent). Perceived health was assessed on a visual analogue scale where 0 represented the worst possible perceived health and 100 the best.

Pre–post-comparison used statistical differences (*p* < 0.05) and correlation with background data (Spearman because the data distributions tended to be slightly skewed), chi-square test *when comparing two categorical variables,* and paired-samples *t*-test determining whether there is a significant difference between the means T1 and T2.

The answers to the open questions were data-analyzed using content analysis as outlined by Graneheim and Lundman (26). Here, the entire data noted by the second and third authors was read and re-read repeatedly. It was then condensed into meaning units, which were sorted into codes containing the core messages. The codes were revised and sorted into preliminary themes and sub-themes. To increase validity, each of the three authors independently read the notes and discussed them until an agreement was reached.

### 2.7 Ethical considerations

Several ethical aspects need consideration in this explorative study. First, the study focused on newly arrived Ukrainians rather than patients. Ukrainians arriving under the mass refugee directive may be vulnerable as they have permission to stay for a certain period and cannot apply for asylum, but by detecting those with high anxiety/stress and perceived illness and offering knowledge and tools to increase perceived health and mental health literacy, one may prevent them from becoming patients. It was also important to consider that questions and discussions during the sessions could raise unwelcome feelings for the participants, reminded of situations that they may wish to avoid. In such cases, our subjects were advised to contact health and mental care and/or follow up online individually with the second and third authors, supervised by the first author. The confidence of the participants was also important. They had consented to participate on the condition of staying anonymous. Thus, any information that might reveal their identity was anonymized.

The strength of the study lies in its integration of a participatory methodology (21), based on a need-based trauma approach, with the experience of refugees in Sweden across diverse domains (Table 1) including psychological and social aspects.

The project was approved before the start by the Swedish Ethical Review Authority (2023-00092-02). All participants gave their written informed consent in accordance with the Declaration of Helsinki.

## 3 Results

### 3.1 Characteristics

The group intervention included eight sets of five group sessions per set (see Table 1), a total of 40 sessions. During the study period, 100 participants participated at the start of the eight sessions. One participant had to be excluded for private reasons. Ninety-nine participants answered the pre-questionnaire before starting (T1).

The results (T1 and T2) of a drop-out analysis (*N* = 42) showed no significant differences between those who answered the questions and those who did not.

Most of the participants were women (83, 83.8%). Half were married (50, 50.5%); approximately one-fifth (23, 23.2%) were unmarried. Just over one-tenth were divorced (15, 15.2%), and about one-tenth (8, 9.0%) were widows. Average age was 45 years (S.D.13.52). The participants had between no and four children (mean 1.51, S.D. 0.973). They had spent on an average of 1 year in Sweden (range 1–17 months) and had in general moved twice due to Migration Agency decisions, but frequency ranged between 1 and 11 times.

The most common mother tongue was Ukrainian (67, 67.8%) followed by Russian (19, 21.2%) and both Ukrainian and Russian (12, 12.1%). Approximately 9 of 10 were born in Ukraine (89, 89.9%) and the others in Russia or the former USSR.

Before arrival, all participants had been working in Ukraine, for example in industry, healthcare, and service. In Sweden, after arrival most had no paid job (72, 80.9%). One-tenth of the employed worked unqualified in hotels, car wash, or restaurants.

### 3.2 Measurement of anxiety/stress and perceived health (T1)

At baseline, T1 (*N* = 93) one-fifth (20, 21.5%) felt no anxiety/stress, two-thirds (62, 66.7%) answered that they felt anxiety/stress to a certain extent, and one-tenth (11, 11.8%) largely felt anxiety/stress before the start. Before the intervention, participants reported the cause of anxiety/stress as follows:

“I’ve lost my job, my home, my financial stability. I’m in a foreign country.”

“I worry about the children and their adaptation.”

“War, anxious about my relatives and not sure about my future, new country, do not understand the mentality and culture and always moving in Sweden.”

“Psychological, mental, somatic (bad health exacerbation of chronic diseases), internal discomfort, a sense of injustice.”

“No opportunity to work and communicate with loved ones.”

“I feel fear for my military brothers.”

The participants with perceived anxiety/stress before the intervention replied that they did the following to feel better:

“I try to distract myself with everyday life or a movie.”

“I communicate with positive people, walk and have fresh air, watch good films.”

“I feel better when I watch TV, cook, do something in the kitchen.”

“Communication with family.”

“I pray, draw and read; I am creative.”

“I try to shout.”

“I eat sweets.”

“I smoke, I listen to music; sometimes I drink alcohol.”

“I go to gym, listen to music, dance, watch videos, bite my nails, cry.”

“Spend more time with kids, we go and see new places.”

At T1 (*N* = 94), the participants noted their present health on a visual analogue scale from 0 to 100, answering on average 63.16 (SD 22.46, range 10–100).

### 3.3 Symptoms of anxiety/stress and perceived health (T1-T2)

Directly after T2, nearly one-third (19, 32.8%) felt no anxiety/stress, six of 10 answered that they felt anxiety/stress to a certain extent and a few (2, 3.4%) answered that they largely felt anxiety/stress direct after. Among those who rated in both T1 and T2, the perceived anxiety/stress was significantly reduced (chi-2 25.53, df = 4, *p* < 001).

After the intervention, the participants perceived anxiety/stress as caused by family, social and economic problems, and adaptation problems such as moving around Sweden as required by the Migration Agency and living in a room with strangers, no work, uncertainty about the future, and relatives in Ukraine.

The participants answered that during the intervention they had received the following knowledge and tools to reduce perceived stress/worries:

“I got contacts I needed, answers to my questions (social, medical), to know the right people, and pleasure from communication.”

“Control of my breathing under stress. I got information from various organizations about their activities: health, safety, and society; what to do act in various problem situations.”

“I have an idea how the health care system works, and the school education system, and where to go in case of need. I also learned to restore my balance with the help of breathing. Thanks to the psychologists for their individual consultation.”

At T2 (*N* = 61), perceived health was on average 71.18 (range 50–100, S.D. 15.89). Perceived health significantly improved between T1 and T2 (*N* = 57, *t* = −3.136, df = 56, *p* < 0.001).

The participants found the controlled breathing exercise very useful in between the meetings, and this was also mentioned in the evaluation.

### 3.4 Spearman’s correlation coefficient

There was a positive significant Spearman correlation between age and number of moves; i.e., the younger had had significantly more moves since arrival (−0.257, *p* < 0.017). There was also a positive significant correlation between the number of months in Sweden and perceived anxiety/stress after intervention (0.325, *p* < 0.017).

There was a negative significant correlation between perceived anxiety/stress before and perceived health before the intervention (−0.589, *p* < 0.001) and perceived health before and anxiety/stress after the intervention (−0.274, *p* < 0.041). There was a positive significant correlation between perceived anxiety/stress before and after the intervention (0.449, *p* < 0.001) and between perceived health before and after the intervention (0.528, *p* < 0.001). There was a negative significant correlation between perceived anxiety/stress and perceived health after the intervention (−0.319, *p* < 0.015).

### 3.5 Group dynamics during eight sets of five intervention sessions

The authors noted that the group dynamics during the total 40 sessions were high; i.e., many of the participants felt trust and asked questions to which they wanted to have answers. This was facilitated by such factors as:

- The participants in most cases knew each other (they were small communities).
- Safety and trust: the rules/policy of the group and the confidentiality of all information were explained before each meeting.
- Primary tension was reduced through initial and concluding breathing practices led by the second and third authors.
- The participants could freely reflect during the meeting (this was facilitated by the open dialogue format of the meetings).
- Active presence of moderators (the second and third authors noted all the questions and answers).
- Emotional expression: The participants openly expressed their feelings and thoughts, which led to a deeper understanding of emotions and contributed to personal growth.
- Support and compassion: The participants offered each other support and compassion. The communal nature of the group allowed people to feel understood and cared for, creating a sense of belonging and reducing feelings of isolation.
- Confidentiality and non-judgment: Confidentiality was strictly enforced within the group, and an atmosphere of non-judgment was cultivated. This allowed participants to share their vulnerabilities without fear of criticism or judgment.
- The participants’ preschool children were given a conducive environment to engage in activities tailored to their developmental needs during the sessions. This fostered a supportive setting that acknowledged the challenges faced by their mothers.

### 3.6 Thematic analysis of participants’ questions

Table 3 presents qualitative results from content analysis of participants’ questions in dialogue with the respective local expert during each session and noted down by the second and third authors. There were 25 sub-themes, and the five themes were as follows: stress and recovery connected to the invasion, health-concerned lack of knowledge on various levels, lack of human rights knowledge, and lack of contact with preschool authorities, and Swedish social legislation was misinterpreted.

**Table 3:** Content analysis, in five themes (columns) and 25 sub-themes of the questions raised by the hälsoskolan participants regarding the EU mass refugee directive (*N* = 100, 8 sets of 5 group sessions per set, a total of 40 sessions). New questions added during the dialogue with each local expert.

1. Stress and recovery connected to the invasion	2. Health concerned lack of knowledge on various levels	3. Split of human rights knowledge	4. Lack of contact with pre- and school authorities	5. Swedish social legislation is misinterpreted
1.1 Lack of personal anxiety management of relocation by the Swedish Migration Agency.	2.1 Language barrier when contacting medical institutions.	3.1 Ways to contact the police are unknown.	4.1 Concerned with bullying at school.	5.1 What the reasons and procedure are for removing children from the family.
1.2. Unaware of intergenerational effects due to psychological trauma.	2.2 Ambulance services and costs for medical service are surprising.	3.2 Interest in ways and means of self-defense in Sweden.	4.2 Wants to know the role of teachers, facilitators, nurses, psychologists, and others.	5.2 What the funeral procedure is for people under mass refugee directive.
1.3 Loss of social roles and existential crisis is easily freezed.	2.3 Women’s health is not prioritized.	3.3 Protection of private property, and territory is central but how?	4.3 Meaning of school attendance as asylum-seeking children have right to attend non-compulsory pre- and school.	5.3 How social assistance and family counselling can be obtained under the directive.
1.4 The problem of divorced spouse and fear for loved ones are escalating.	2.4 Vaccination of adults and children differs between the countries.	3.4 Want knowledge about criminal liability and age.	4.4 Teenagers’ working age and kind of work.	5.4 What support a family violence victim can receive.
1.5 Fear to returning home competes with wish to stay.	2.5 Difficulty to contact dentist.	3.5 Peculiar rules for Ukrainian refugees with EU mass directive.	4.5 How extracurricular activities for children are organized.	5.5 Facts about the spread of prostitution, drug addiction, alcoholism.

## 4 Discussion

The study aimed to investigate the feasibility and effectiveness of a short, trauma-focused group intervention (in Swedish “*hälsoskola*”) for Ukrainian-speaking refugees. A mixed-methods design and participatory methodology and evaluation were used to examine how Ukrainian refugees perceived their health before and after the intervention. The hypothesis was confirmed. The short group intervention had a significantly positive impact on perceived health, which significantly reduced perceived stress/anxiety and led to symptom reduction. The results of correlations can be interpreted in terms of stability of stress/anxiety and perceived health, but there is a need for longitudinal evaluation to measure the present state of reduction of mental health problems. The participants also performed brief, controlled breathing exercises to reduce bodily stress on their own in between the meetings. According to the five themes (Table 1), the findings are in line with the ADAPT theoretical model developed by Silove (2) and a participatory methodological approach (21). The dialogue between the participants’ needing knowledge and the local expert’s direct answers may have been a key to increased perceived health and mental health literacy. Qualitative results from content analysis of participants’ questions raised in dialogue with their local expert during the session add supporting details regarding their limbo situation and separation from relatives; while the knowledge gained from the local expert increased their health and mental health literacy and they wanted to learn more. However, due to their perceived limbo situation, unemployment and separation from relatives, they wanted to learn more how to sustain control over perceived anxiety/stress in a follow up (hälsoskola) if possible in the future.

Given the unique demographic composition of the refugee population from Ukraine, characterized predominantly by mothers with children and bereft of spousal support, the study implemented a multifaceted intervention strategy to address distinct needs. Recognizing the intricate challenges faced by these mothers, particularly in the absence of paternal support, the study adopted a need-based approach to trauma during the sessions.

### 4.1 Discussion of results

Research on the development and course of post-traumatic stress disorder after a single potentially traumatic experience shows that approximately one-third suffer from PTSD (5), but many participants probably have several traumas, so the number can be higher. Swedish healthcare lacks resources to accommodate a third of the Ukrainian’s estimated need for health and mental health literacy. For these reasons, health literacy should be central to refugee reception. A study by Wångdahl et al. (27) showed that a substantial number of refugees in Sweden are incompletely health literate and reported less-than-good health and impaired wellbeing; or that the informants had avoided seeking healthcare. Al-Adhami et al. (28) showed in a study in Sweden that extended health communication included in the civic orientation course improves health literacy of newly settled refugees. Thus, preventive intervention is of significance and may increase social inclusion and integration (8) as well as mental health support, for Ukrainian survivors under the EU mass refugee directive. The present short intervention for reducing stress and anxiety may also reduce a second emotional wave when a new trauma arises with the continuation of war following the Russian invasion. Stress-induced disorders, including depression and anxiety, are common and cannot wait until the war is over.

Family coping resources are diminished by long forced separation, physical injury, and mental illness (e.g., negative thoughts). Children will forget their separated parent (often their father) which may hamper their development and trust (13). Findings indicate the significance of engaging families in planning and skill-building to support healthy communication despite separation due to war. Given these potential risks of developing social, marital, and health problems among Ukrainian soldiers, it is important to understand protective factors that may reduce these outcomes and community reintegration into society (28). The integrated approach to reintegration practiced by the International Organization for Migration (29) recognizes that the complex process of reintegration requires a holistic and a needs-based response at the individual, community, and structural levels.

One potential protective factor may be social support through communication (e.g., phone, WhatsApp, email, and video calls) with a separated significant person or loved one. This may protect against the psychosocial stressors of military deployment. Furthermore, another protective factor is the increase in our subjects’ perceived health and mental health literacy through the participatory approach (21). To work with the topic of healthy communication in the field demands knowledge and experience, and this is supported by the WHO (4), statement that “addressing these determinants and enhancing the health of migrants, refugees and other displaced populations are essential goals for global health and sustainable development” (page 1).

The present study shows a need for a scientific and cultural methodological participatory approach (21) to increase perceived health and mental health literacy, by developing resilience through post-migration growth while reducing suffering and minimizing disability for survivors of traumatic life events (30). This should be a priority in the negative and positive components of family communication during displacement, promoting repatriation when possible.

### 4.2 Discussion of qualitative design

Below, we will discuss the significance of using qualitative methods, as we explored studying a new target group, and the aim has not been studied before.

#### 4.2.1 Subjectivity and power

According to Malterud (31), that researchers do not influence content is a naïve perception, which is rejected in modern scientific theory. A qualitative design seeks to collect knowledge from the participants’ own life experiences and is therefore suitable for exploring perceptions, attitudes, and experiences. The second and third author came from the same cultural and language background as the participants, and had field related expertise which increased the contact and trust with the participants. An interpreter was usually in the room during the group meetings and the authors mentioned could check the quality of the translation and explore if they found the interpretation difficult to understand.

#### 4.2.2 Categories

In the qualitative content analysis, the sub-themes were developed from the codes and all three authors independently coded, discussed, and reached consensus during their frequent meetings. Further subcategorization according to, for example, social determinants was performed. However, the participants’ stress and worries due to the EU mass refugee directive may have had greater weight than other background factors (education, gender, and socioeconomic status).

### 4.3 Strengths and limitations

The strength of the study is that the second and third authors have the same background and languages (Ukrainian and Russian), were educated in psychology mainly in the home country, and were supervised by the first author. They telegrammed the participants the day before the group meeting, and this promoted trust. The participants had high perceived trust in them but less in local and national authorities. The participants were given PowerPoint presentations translated by the second and third authors during each group meeting, and this may also have increased health and mental health literacy.

Further, to evaluate qualitative studies, an assessment of their reliability is central (trustworthiness). Graneheim and Lundman (26) take up three different aspects of trustworthiness as central: credibility, dependability, and transferability. Credibility concerns whether the chosen design is suitable to answer the aim and whether the analysis was performed expediently. We used qualitative content analysis as suitable for our aim, and there was an ongoing collaboration between the three authors, with triple coding and comprehensive discussion of categorization. To increase validity, each of the three authors independently read the notes and discussed them until an agreement was reached.

Dependability refers to changes in the material and analysis over time. As the project lasted only 11 months, our data are presumed to reduce the risk of changes in the analysis.

Transferability refers to whether the material can be transferred to other groups and contexts. To validate transferability, we informed newcomers about our experience in the project, and many were interested in participating. The validity of the assessment of mental health seems to be promising in the explorative study as pre- and post-assessments of stress/anxiety and perceived health showed improvements, which have also been shown in earlier studies by the first author and colleagues (8).

Limitations were the geographical distance between the participants and the three authors. This was solved by using Teams during four of the five meetings. However, Teams was not always optimal. Furthermore, due to the project’s time limitation we were unable to follow up on the perceived health and mental literacy after 6 months or longer or to assess whether the participants could find a job related to their increased perceived health and mental literacy. Therefore, we were unable to study the theory of “the healthy migrant effect.” This states that the person who emigrates shows better health both compared to the population in the home country and to the majority population of the new country upon arrival. That there is then a deterioration in health indicates that something happens during or after the migration that affects the individual’s health negatively (32). Further, as the study was explorative the limitations regarding representativity and social desirability were not feasible to study.

### 4.4 Implications

According to Kumar et al. (1), migrant health research in the Nordic countries has yet not been prioritized and health policies and practice, especially long-term national plans, often exclude migrants and implications for policy and practice that could enable societal conditions to reduce avoidable health inequalities.

Up until now, and to our knowledge, Swedish refugee reception (The Swedish Migration Agency, Ministry of Foreign Affairs, SIDA) has rarely targeted these problems in the repatriation process and community reintegration (33). There is an urgent need to develop an intervention to improve partnership quality and reduce the stressors that Ukrainian displaced partners and their children face. We do not know when the invasion by Russia will be stopped. What is needed is to examine whether couples’ perceptions of positive or negative communication moderate the relationship between how frequently they communicate during separation and their mental distress.

### 4.5 Future research

According to the EU mass refugee directive on the time limit for stay (4 March 2025), the Tidal Agreement emphasizes repatriation and the build-up of infrastructure in Ukraine, highlighting trust and cohesion between separated couples to reduce mental distress, is also of importance. However, to our knowledge, this has yet not been studied. Further, a planned explorative study will approach collaboration, train-the-trainer, and dialogue online and as soon as real life permits, exchange with trauma centers in Ukraine.

## 5 Conclusion

The present co-creation short group intervention with separated Ukrainian families seems to be feasible and effective for increasing health and mental health literacy. It will promote prevention options for future repatriation. A better understanding of the individual and collective needs of newcoming Ukrainians will allow specialists in many areas to help people faster and better, also considering national characteristics. It is important as many Ukrainians have already become part of Swedish society.

## Data availability statement

The raw data supporting the conclusions of this article will be made available by the authors, without undue reservation.

## Ethics statement

The studies involving humans were approved by Swedish Ethical Review Authority (2023-00092-02). The studies were conducted in accordance with the local legislation and institutional requirements. The ethics committee/institutional review board waived the requirement of written informed consent for participation from the participants or the participants’ legal guardians/next of kin because informed consent is obligatory when you apply for ethical approval. Written informed consent was obtained from the individual(s) for the publication of any potentially identifiable images or data included in this article.

## Author contributions

SE: Formal analysis, Investigation, Validation, Writing – original draft, Writing – review & editing. OG: Formal analysis, Investigation, Validation, Writing – review & editing. YS: Formal analysis, Investigation, Validation, Writing – review & editing.
